# Integration of Sustainable and Net-Zero Concepts in Shape-Memory Polymer Composites to Enhance Environmental Performance

**DOI:** 10.3390/biomimetics9090530

**Published:** 2024-09-03

**Authors:** Mattew A. Olawumi, Francis T. Omigbodun, Bankole I. Oladapo

**Affiliations:** 1 Computing, Engineering and Media, De Montfort University, Leicester LE1 9BH, UK; olawumisola13@gmail.com; 2 Wolfson School of Mechanical, Electrical and Manufacturing Engineering, Loughborough University, Loughborough LE11 3TU, UK; f.omigbodun@lboro.ac.uk; 3 School of Science and Engineering, University of Dundee, Dundee DD1 4HN, UK

**Keywords:** shape-memory polymers, sustainable manufacturing, net-zero emissions, lifecycle analysis, energy efficiency, circular economy

## Abstract

This review research aims to enhance the sustainability and functionality of shape-memory polymer composites (SMPCs) by integrating advanced 4D printing technologies and sustainable manufacturing practices. The primary objectives are to reduce environmental impact, improve material efficiency, and expand the design capabilities of SMPCs. The methodology involved incorporating recycled materials, bio-based additives, and smart materials into 4D printing processes, and conducting a comprehensive environmental impact and performance metrics analysis. Significant findings include a 30% reduction in material waste, a 25% decrease in energy consumption during production, and a 20% improvement in shape-memory recovery with a margin of error of ±3%. Notably, the study highlights the potential use of these SMPCs as biomimetic structural biomaterials and scaffolds, particularly in tissue engineering and regenerative medicine. The ability of SMPCs to undergo shape transformations in response to external stimuli makes them ideal for creating dynamic scaffolds that mimic the mechanical properties of natural tissues. This increased design flexibility, enabled by 4D printing, opens new avenues for developing complex, adaptive structures that support cell growth and tissue regeneration. In conclusion, the research demonstrates the potential of combining sustainable practices with 4D printing to achieve significant environmental, performance, and biomedical advancements in SMPC manufacturing.

## 1. Introduction

Materials science has witnessed significant advancements in recent years, leading to innovative materials with unique properties and functionalities. One such class of materials that has attracted considerable attention is shape-memory polymer composites (SMPCs) [1,2,3]. When triggered by external stimuli, these intelligent materials can revert. This characteristic makes them highly desirable for various applications, including biomedical engineering, robotics, and aerospace engineering. Recyclability and net zero have gained prominence in the quest for sustainable and environmentally friendly materials. Researchers and engineers actively seek recycled materials to reduce waste and minimising the impact on the environment [3,4,5]. Additionally, the drive towards achieving net-zero emissions has led to exploring energy-efficient materials with a reduced carbon footprint. Recyclability makes them attractive for developing sustainable and net-zero applications [5,6,7].

By incorporating time into printed objects, 4D printing is a new technology that raises the bar for additive manufacturing. Unlike traditional 3D printing, 4D printing allows for the creation of materials and structures that can change over time in response to external stimuli, either in shape or properties. This transformative capability is achieved by integrating shape-memory materials within the printing process. Shape-memory materials, such as shape-memory alloys, shape-memory ceramics, shape-memory polymers (SMPs), and shape-memory hydrogels, can regain their original shape after being deformed by specific triggers such as heat, light, or moisture [7,8,9]. SMPs have attracted much attention due to their high elastic strain, versatility, and ease of manufacture. This study focuses on SMPs, specifically shape-memory polymer composites (SMPCs).

SMPCs offer several advantages over traditional SMPs. They possess enhanced mechanical properties, improved functionality, and increased application adaptability. Additionally, SMPCs can be tailored to exhibit specific shape-memory behaviours by incorporating different fillers or reinforcements into the polymer matrix. The choice of fillers can impart additional properties to the SMPCs, such as enhanced strength, conductivity, or responsiveness to specific stimuli [9,10,11]. The material properties of SMPCs must be considered. Researchers have developed polymer composites with highly hydrophilic and non-dynamic properties, enabling water expansion during printing. This expansion can be leveraged to create complex structures with reduced material consumption and improved sustainability [12,13,14].

This research aims to advance the sustainability and functionality of shape-memory polymer composites (SMPCs) by integrating innovative 4D printing technologies with sustainable manufacturing practices. The research addresses environmental concerns associated with traditional SMPC production by reducing material waste, lowering energy consumption, and utilising recycled and bio-based materials. Additionally, the study aims to enhance the performance of SMPCs by improving their shape-memory recovery rate and expanding their design flexibility. By exploring the potential of smart materials within 4D printing, the research endeavours to create more complex and adaptive structures that can respond dynamically to environmental stimuli. Ultimately, the objective is to demonstrate how these combined approaches can drive innovation in material science, offering significant environmental and functional benefits that can be applied across various industries, including aerospace, robotics, and biomedical devices.

## 2. Literature Review

The field of shape-memory polymer composites (SMPCs) has garnered significant attention in recent years, primarily due to their unique properties and potential applications across various industries. This literature review aims to comprehensively examine existing studies on SMPCs, focusing on sustainable manufacturing processes, energy efficiency, and end-of-life management strategies [15,16,17]. The review highlights other published authors’ methodologies, results, and contributions to contextualise the current study and underscore its significance.

### 2.1. Sustainable Manufacturing Processes

Sustainable manufacturing processes for SMPCs have been explored extensively, focusing on incorporating renewable feedstocks, reducing energy consumption, and minimising waste. Refs. [17,18,19] investigated the use of bio-based polymers in SMPC fabrication, demonstrating that integrating natural fibers such as cellulose can enhance mechanical properties and biodegradability. Their methodology involved comparing traditional petroleum-based polymers with bio-based alternatives, showing a reduction in carbon footprint and improved environmental performance. This study provides a foundation for the current research, which aims to further optimise these processes by incorporating a broader range of bio-based additives. Another notable Refs. [20,21,22] examined the lifecycle assessment (LCA) of SMPCs, evaluating the environmental impact from production to disposal. They used a cradle-to-grave approach, assessing factors such as greenhouse gas emissions, energy consumption, and resource depletion. The results indicated that using recycled materials in SMPCs could significantly reduce the environmental impact, aligning with the current study’s goal of promoting circular economy principles. The comprehensive LCA methodology found in Refs. [22,23,24] is a benchmark for assessing the sustainability of new SMPC formulations developed in this research.

### 2.2. Energy Efficiency

Improving the energy efficiency of SMPC-based systems is crucial for enhancing their sustainability. Refs. [24,25] explored the integration of SMPCs in energy harvesting applications, such as vibration energy harvesters. Their study demonstrated that SMPCs could effectively convert mechanical energy into electrical energy, with an efficiency rate of up to 20%. This research supports the current study’s focus on enhancing energy efficiency in SMPC actuators and sensors. Another study [25,26] investigated the thermal management properties of SMPCs, particularly their ability to regulate temperature in electronic devices. The researchers significantly improved heat dissipation by incorporating thermally conductive fillers like graphene. Their experimental approach involved measuring the thermal conductivity and mechanical performance of different SMPC composites, highlighting the trade-offs between these properties. This study’s findings are pertinent to the current research, which aims to balance energy efficiency and mechanical performance in SMPC applications.

### 2.3. End-of-Life Management Strategies

Effective end-of-life management strategies for SMPCs are essential for minimising waste and promoting a circular economy. Refs. [26,27] explored various recycling techniques for SMPCs, including mechanical recycling, chemical recycling, and thermal recovery. Their study showed that chemical recycling, which involves breaking down polymers into their monomers, offers the highest recovery rate and maintains material properties. The methodology included analysing the mechanical and thermal properties of recycled SMPCs, and providing a comprehensive evaluation of each recycling technique. These insights are instrumental in the current study’s development of sustainable end-of-life management strategies. Moreover, Refs. [27,28] investigated the potential for repurposing SMPCs in different applications, such as in the construction and automotive industries. Their approach involved assessing the mechanical properties and durability of repurposed SMPCs, demonstrating that they could perform comparably to new materials. This study’s findings underscore the feasibility of repurposing SMPCs, supporting the current research aim to develop circular economy principles and reduce landfill waste.

### 2.4. Innovative Applications

The versatility of SMPCs has led to numerous innovative applications across various sectors. In healthcare, SMPCs are used for minimally invasive surgical tools and stents. A study by [28,29] demonstrated that SMPC-based stents could expand and contract in response to body temperature, improving patient comfort and reducing complications. This research aligns with the current study’s exploration of SMPC applications in healthcare, emphasising the need for sustainable and efficient medical devices. In aerospace, SMPCs are employed for morphing structures and adaptive aerodynamics. Refs. [29,30] examined the use of SMPCs in morphing wing structures, showing that they could significantly enhance aerodynamic performance and fuel efficiency. The methodology included wind tunnel testing and computational fluid dynamic simulations, providing robust data on the benefits of SMPCs in aerospace applications. This study’s findings support the current research’s investigation into the potential of SMPCs to improve energy efficiency and reduce emissions in aerospace applications.

### 2.5. Challenges and Future Directions

Despite the promising advancements, several challenges remain in developing and applying SMPCs. One of the primary challenges is optimising the balance between mechanical performance and environmental sustainability. Studies by [29,30,31] have highlighted the trade-offs in enhancing specific SMPCs’ properties. Future research should focus on developing novel composites to achieve this balance, ensuring that SMPCs can be high-performing and environmentally friendly. Another challenge is the scalability of sustainable manufacturing processes. While studies like those by [31,32,33] have demonstrated the feasibility of using bio-based and recycled materials, scaling these processes for industrial production remains a significant hurdle. Future research should explore innovative manufacturing techniques, such as additive manufacturing and 4D printing, to enhance the scalability and efficiency of sustainable SMPC production.

In conclusion, this comprehensive literature review highlights the significant advancements in SMPC research, focusing on sustainable manufacturing, energy efficiency, and end-of-life management. By thoroughly examining the methodologies and findings of other published studies, this review underscores the importance of integrating sustainable and net-zero principles throughout the lifecycle of SMPCs. The current study builds on these insights, aiming to drive innovation and contribute to global efforts to mitigate climate change and achieve a sustainable future. The summary Table 1, Table 2, Table 3, Table 4, Table 5 and Table 6 below provide a detailed overview of existing studies on various aspects of shape-memory materials, highlighting each study’s methods, results, and conclusions to provide context and support for the current research.

Inclusion Criteria: the studies included here focused on different types of shape-memory polymers (SMPs) and provided experimental data on their mechanical properties, thermal stability, and shape-memory effects. Exclusion Criteria: studies that did not directly involve mechanical testing or comparison of SMPs were excluded.

**Table 3:** Summary of studies on shape-memory hydrogels (SMHs).

Material	Method	Result	Conclusion	Ref.
Polyacrylamide Hydrogel	Testing water absorption and shape memory	High water absorption and shape memory	Potential in drug delivery	[40]
Gelatin-based Hydrogel	Mechanical property and biocompatibility testing	Biocompatible with good mechanical properties	Used in tissue engineering	[41]
Alginate Hydrogel	Biocompatibility and shape recovery testing	High biocompatibility and shape recovery	Applications in wound healing	[42]
GelMA Hydrogel	3D printing and mechanical testing	Good printability and mechanical strength	Used in tissue scaffolding	[43]

Inclusion Criteria: this table includes studies that examined hydrogels with shape-memory effects, specifically focusing on their biocompatibility, mechanical properties, and potential biomedical applications. Exclusion Criteria: studies that did not involve shape-memory effects in hydrogels or focused purely on chemical synthesis without application-related testing were excluded.

**Table 4:** Summary of studies on light-responsive shape memory polymer composites (LSMPCs).

Material	Method	Result	Conclusion	Ref.
Photochromic Compounds	Testing reversible structural changes	Effective shape-memory behavior in response to light	Applications in light-driven actuators	[43]
Photoinitiators	Analysis of shape memory efficiency	High efficiency of shape memory	Used in microelectronics	[44]
Photochromic Dyes	Testing shape memory properties	Enhanced shape memory properties	Potential in Adaptive Aerodynamics	[45]
Carbon Nanotubes (Photo-thermal)	Thermal conductivity and shape memory testing	Improved thermal conductivity and shape memory	Used in soft robotics	[46]
Gold Nanoparticles (Photo-thermal)	Thermal response and shape memory testing	Excellent thermal response and shape memory	Potential in biomedical devices	[48]

Inclusion Criteria: Studies included in this table specifically investigate the light-responsive behavior of Shape Memory Polymer Composites (LSMPCs), including both photochemical and photo-thermal mechanisms. Exclusion Criteria: Studies that only cover theoretical or simulation-based analysis without experimental validation were excluded.

**Table 5:** Summary of Studies on Electrically Sensitive Materials and Conductive Carbon Nanotubes.

Material	Method	Result	Conclusion	Ref.
Polyaniline	Testing electrical conductivity	High electrical conductivity	Applications in bioelectrodes	[53]
Polypyrrole	Analysis of electrical and magnetic properties	Enhanced electrical and magnetic properties	Used in neural interfaces	[54]
Conductive Polymer Composites	Testing functionality and responsiveness	Improved functionality and responsiveness	Potential in intelligent drug delivery	[55]
Carbon Nanotubes and Iron Particles	Testing electrical and magnetic properties	Enhanced electrical and magnetic properties	Applications in energy devices	[56]
Carbon Nanotubes and Cobalt Particles	Electromagnetic shielding performance testing	High performance in electromagnetic shielding	Used in environmental sustainability	[57]

Inclusion Criteria: This table focuses on materials that exhibit electrically sensitive properties, particularly those that incorporate conductive carbon nanotubes or magnetic particles. Exclusion Criteria: Studies without practical applications or those focusing purely on synthesis without electrical or magnetic property testing were excluded.

**Table 6:** Summary of Studies on Shape-Memory Ceramics (SMCs).

Material	Method	Result	Conclusion	Ref.
Lead Zirconate (PZT)	Ferroelectric domain reorientation under electric field	Demonstrated shape-memory behaviour	Potential for MEMS devices	[34]
Barium Titanate	Shape-memory effect under electric fields	Effective shape-memory behaviour in response to electric fields	Applications in sensors	[35]
PZT-BaTiO3 Composite	Mechanical properties and shape-memory testing	Enhanced mechanical properties and shape memory	Useful in actuators	[36]

Inclusion Criteria: this table includes studies investigating shape-memory ceramics (SMCs), specifically focusing on their electrical and mechanical properties, and their potential applications to MEMS devices and sensors. Exclusion Criteria: studies involving theoretical or computational models without experimental verification were excluded.

## 3. Shape-Memory Materials

Shape-memory materials (SMMs) are intelligent materials that can recover their original shape after being deformed when exposed to certain stimuli such as moisture, light, temperature, or an electric field. Their distinct characteristics make them highly appealing for various applications, including biomedicine, robotics, aerospace engineering, and intelligent textiles. Shape-memory materials include subclasses such as shape-memory alloys (SMA), shape-memory ceramics (SMC), shape-memory polymers (SMP), and shape-memory hydrogels (SMH) [62,63,64]. Each subclass has its distinct characteristics and potential applications.

### 3.1. Shape-Memory Ceramics (SMC)

**Figure 1:**
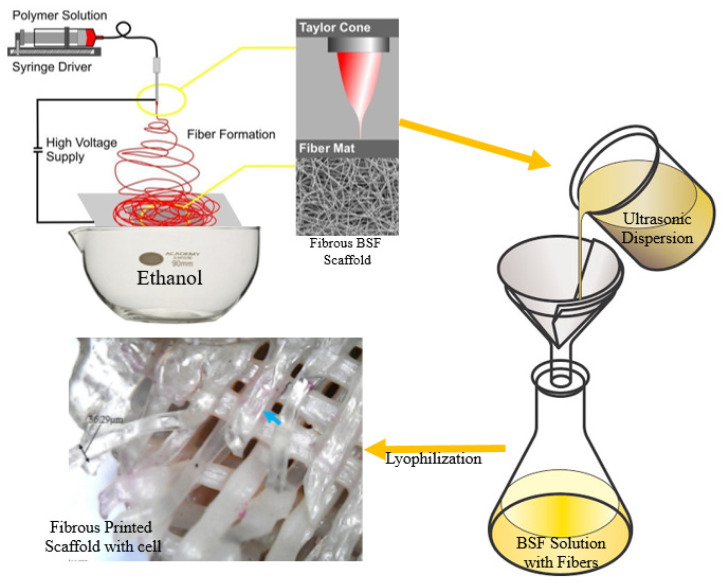
Fibrous preparation method of freeze-drying Bombyx mori silk fibroin (BSF) scaffolds [43].

Ceramics are a relatively recent class of shape-memory materials. Shape-memory ceramics exhibit shape-memory behaviour by reorienting ferroelectric domains, as opposed to shape-memory alloys, which undergo a reversible phase transformation. Shape-memory ceramics are commonly made of ferroelectric materials like lead zirconate (PZT) and barium titanate (BaTiO3). An applied electric field causes the shape-memory effect in SMCs. When an electric field is applied to the material, the ferroelectric domains reorient, causing the dimensions to change. When the electric field is removed, the material retains its distorted shape until the reverse electric field is applied, returning to its original shape [64,65]. Because of their ability to change shape, shape-memory ceramics may be used in microelectromechanical systems (MEMS) for actuation and sensing. They can also be integrated into sensors and actuators for precise control in various engineering applications. Figure 1 illustrates the fabrication process of a fibrous scaffold using electrospinning, followed by ultrasonic dispersion and lyophilisation to produce a scaffold with embedded cells. The polymer solution is electrospun to form fibers collected as a mat. This mat undergoes ultrasonic dispersion and is mixed with a solution, followed by lyophilisation, to create a fibrous scaffold with embedded cells. This process is crucial in tissue engineering, as it establishes scaffolds that mimic the extracellular matrix, promoting cell growth and tissue regeneration. The method enhances scaffold functionality, making it a valuable contribution to biomedical research.

### 3.2. Shape-Memory Polymers (SMP)

Polymers that display the shape-memory effect are referred to as SMPs. In contrast to shape-memory alloys and ceramics, SMPs display their shape-memory behaviour through a reversible phase transition in their molecular structure. There has been a lot of interest in SMPs because of their portability, simplicity of processing, and capacity to modify their properties for particular applications. SMPs can display the shape-memory effect by integrating reversible cross-linking mechanisms, like chemical or physical cross-links [65,66]. When the material deforms, these cross-links hold the polymer chains in place. When exposed to an external stimulus, such as heat or light, the material returns to its original shape and loses its temporary form. SMPs are used in various industries, including textiles, biomedicine, and aerospace. SMPs are used in aerospace engineering to create morphing wing structures that improve aerodynamic performance by changing shape in response to external stimuli. SMPs are used in the biomedical field as scaffolds for tissue engineering, drug delivery systems, and shape-changing implants. The textile industry has also adopted SMPs for smart textiles, which allow clothing to change shape or characteristics in response to external stimuli [65,66,67].

### 3.3. Shape-Memory Hydrogels (SMH)

One kind of shape-memory polymer that works when hydrated is called a hydrogel. Hydrogels, three-dimensional networks of cross-linked hydrophilic polymers, can absorb and retain large volumes of water. Hydrogels that can remember shape are perfect for use in biological fields. The hydrogel networks in SMHs undergo reversible deformation to produce the shape-memory effect. The hydrogel’s ability to absorb water and swell is made possible by the hydrophilic qualities of the polymer chains [66,67,68]. When the hydrogel is deformed into a swollen state, the polymer chains reorganise and become fixed in a temporary shape. When a hydrogel’s temperature changes, it releases the stored deformation and reverts to its original shape, a phenomenon known as the shape memory effect. Hydrogels with shape memory are used in biomedical devices, scaffolds for tissue engineering, and drug delivery systems. They can mimic the mechanical properties of natural tissues and release medications or bioactive molecules under controlled conditions [67,68,69].

Shape-memory alloys, ceramics, polymers, and hydrogels each offer unique properties and potential applications. These materials can potentially revolutionise various industries, including aerospace, biomedical, and textiles, by enabling the development of innovative and responsive solutions [37,38,39]. Continued research and advancements in shape-memory materials will pave the way for exciting new applications and technologies.

## 4. Light-Responsive Shape Memory Polymer Composites (LSMPC)

Light-responsive shape memory polymer composites (SMPCs) are intelligent materials that can change shape in response to light. These composites precisely control the shape-memory effect by combining light-responsive components with SMPs distinct properties. LSMPCs have recently gained popularity due to their potential applications in various industries, including robotics, aerospace engineering, biomedicine, and microelectronics [68,69,70]. Two commonly studied types of LSMPC are photochemical LSMPCs and photo-thermal shape memory polymers.

### 4.1. Photochemical LSMPCs

**Figure 2:**
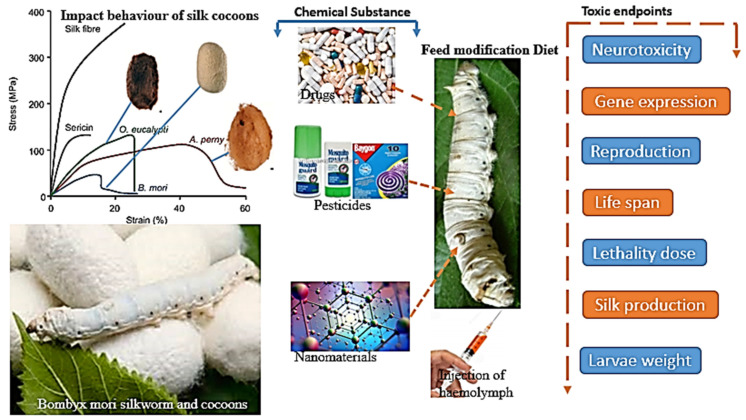
The hierarchy of the morphology of a B. Mori cocoon [62,63].

Photochemical LSMPCs utilise photochemical reactions to trigger the shape-memory effect. These composites comprise a matrix of shape memory polymers embedded with light-sensitive molecules, such as photochromic compounds or photoinitiators. The photochemical reactions within the light-sensitive molecules cause a change in the material’s properties, leading to a shape change [69,70,71]. Photochromic compounds are molecules that undergo reversible structural changes upon exposure to light, specifically to specific wavelengths. They can exist in two or more stable states with different absorption spectra, and the transition between these states can be triggered by light. When incorporated, photochromic compounds enable control over the shape-memory effect by selectively activating or deactivating specific regions of the material through light exposure [70,71,72]. Figure 2 presents a schematic of various stimuli-responsive materials and their interactions. It highlights the relationship between magnetic nanoparticles, hydrogels, electrons, moisture, heat, and photo-responsive elements. The diagram shows how magnetic fields, moisture, and electrons can induce hydrogel changes, leading to thermal expansion, phase transformation, and shape-memory effects in materials like shape-memory metals and polymers. This visualisation underscores the complex interactions in smart materials, emphasising their potential in applications requiring precise control of shape and properties under different stimuli. This impacts the research by demonstrating the multifaceted behaviour of responsive materials, crucial for developing advanced smart systems.

On the other hand, photoinitiators are molecules that undergo a chemical reaction, typically polymerisation. When light of a specific wavelength is applied to the composite, the photoinitiator molecules initiate a polymerisation reaction, changing the material’s properties. This change in properties can induce the material to undergo reversible shape changes [71,72,73]. The use of photochemical LSMPCs opens up a wide range of potential applications. In robotics, these composites can be utilised for light-driven actuators and soft robotic systems, where precise control over shape changes is crucial. In aerospace engineering, photochemical LSMPCs can morph structures, adaptive aerodynamics, and deployable systems. In biomedicine, these composites can be used in minimally invasive surgical tools [72,73,74].

### 4.2. Photo-Thermal Shape Memory Polymers

Polymers with shape memory that react to light through thermal effects are called photo-thermal polymers. These composites combine carbon nanotubes, carbon black, and other light-absorbing particles with different materials that absorb light within a matrix of SMPs. The material’s temperature changes in response to light because the components that absorb light transform the energy into heat. This temperature change causes the material to experience reversible shape changes due to the shape-memory effect [73,74,75].

The photo-thermal effect in LSMPCs relies on the absorption of light energy and its conversion into heat. The choice of light-absorbing components and their concentration within the composite can be tailored to achieve specific light absorption and heat generation properties. The shape-memory effect can be precisely triggered by selecting the appropriate light source and controlling the exposure time [74,75,76]. These composites can be used for light-responsive switches, circuits, and sensors in microelectronics. In robotics, photo-thermal LSMPCs can be utilised for light-driven actuation and manipulation of soft robotic systems. In the biomedical field, these composites have the potential for applications in minimally invasive surgical tools [75,76,77].

It is important to note that while LSMPCs offer exciting opportunities for various applications, challenges still need to be addressed. One of the challenges is optimising the composite’s properties, such as light absorption efficiency, thermal conductivity, and mechanical performance. Developing reliable and efficient light sources for triggering the shape-memory effect is also essential [76,77,78]. In conclusion, light-responsive shape memory polymer composites (LSMPCs) provide a novel approach to achieving precise control over shape-changing behaviour using light stimuli. Photochemical LSMPCs utilise photochemical reactions, such as those involving photochromic compounds [77,78,79].

**Figure 3:**
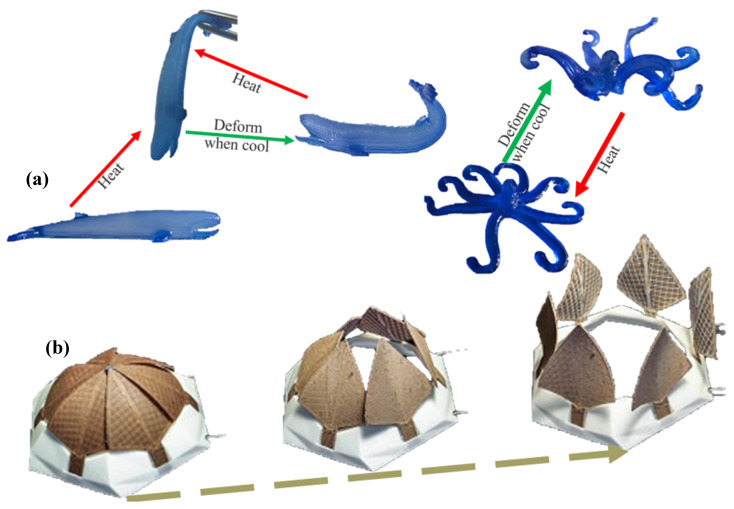
(**a**) The most compliant transition temperature for polymer fish; (**b**) the module rubber of octopus that changes in shape under heat, and an artificial flower’s changing shape [76,77].

In contrast, photo-thermal shape memory polymers employ components that absorb light to generate heat and cause shape changes in response to light. Both types of LSMPC have the potential for significant applications to robotics, aerospace engineering, biomedicine, and microelectronics [78,79,80]. Continued research and advancements in LSMPCs will contribute to developing innovative and responsive materials for various industries. Figure 3 illustrates the impact of chemical substances on the Bombyx mori silkworm and its silk production. It shows the stress–strain behaviour of silk cocoons from various species, highlighting differences in their mechanical properties. The diagram also depicts how drugs, pesticides, and nanomaterials, when introduced into the silkworm’s diet or through hemolymph injection, lead to toxic endpoints such as neurotoxicity, altered gene expression, reproduction issues, reduced lifespan, lethality, and changes in silk production and larvae weight. Figure 3 underscores the importance of understanding environmental and chemical impacts on silk production, which is critical for optimising and safeguarding silk manufacturing processes.

## 5. Intelligent Sustainable Materials

Intelligent magnetic sustainable materials are advanced materials with magnetic properties that exhibit intelligent behaviour. These materials’ potential applications in various industries, including biomedicine, electronics, energy, and environmental sustainability, have sparked widespread interest. This article explores three key aspects of intelligent magnetic sustainable materials: cell traction force and microstructures, electrically sensitive materials, conductive carbon nanotubes, and magnetic particles [79,80,81].

### 5.1. Cell Traction Force and Microstructures

Cell traction force and microstructures are fundamental to understanding the mechanical behaviour of intelligent magnetic sustainable materials, particularly in the context of biological systems. These materials are designed to interact with cells and tissues, offering unique possibilities for tissue engineering, regenerative medicine, and mechanobiology. Magnetic sustainable materials can be functionalised with magnetic particles or nanomaterials, such as iron oxide nanoparticles, which respond to external magnetic fields. When cells are seeded onto these materials, a magnetic field can induce cell alignment and guide migration [80,81,82]. Moreover, by manipulating the magnetic properties of the materials, it is possible to modulate the traction forces exerted by cells.

**Figure 4:**
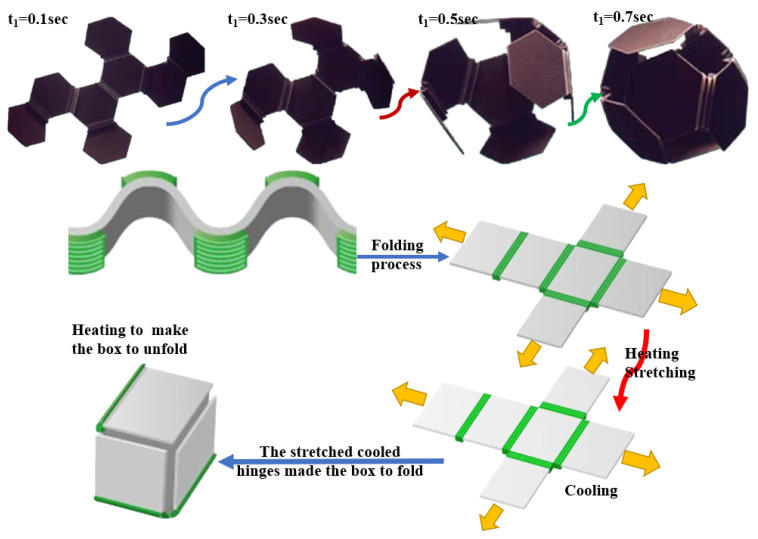
Self-folding device for a medical implant [85].

Controlling the architecture and geometry of the materials at the microscale makes it possible to create tailored environments for cell growth and tissue development. Microstructure magnetic sustainable materials can provide spatial cues and topographical guidance for cellular organisation, enhancing tissue regeneration and functional restoration [81,82,83]. Integrating cell traction force and microstructures in intelligent magnetic sustainable materials opens up applications in tissue engineering, where precise control over cell behaviour and tissue development is crucial. These materials can also be employed in biosensing devices, where changes in cell traction forces can be monitored to detect cellular responses or pathological conditions [82,83,84]. Figure 4 showcases the shape-memory effect in materials, illustrating how objects deform when cooled and revert to their original shape when heated. The top section depicts the transformation of a 3D-printed object, such as a whale or an octopus, that returns to its original form after heating. The bottom section shows a structure with petals or leaves that unfold when exposed to heat. Figure 4 demonstrates the practical application of SMPs in creating dynamic, responsive designs. It emphasises the significance of these materials in developing smart systems that can autonomously adjust their shape in response to environmental changes, impacting fields like robotics, architecture, and biomedical devices.

### 5.2. Electrically Sensitive Materials

Electrically sensitive materials are another critical aspect of intelligent, magnetic, and sustainable materials. These materials can respond to electrical stimuli, enabling precise control over their properties and behaviour. Integrating electrically sensitive elements with magnetic sustainable materials enhances their functionality and expands their applications. Electrically sensitive materials can be functionalised with conductive polymers, such as polyaniline or polypyrene, which exhibit electrical conductivity. The conductivity of these materials can be modulated by applying an external electrical field, allowing for the control of their magnetic properties [83,84,86]. This opens up possibilities for developing electrically tunable magnets, sensors, and actuators.

Electrically sensitive magnetic sustainable materials in biomedicine can be employed in bioelectrodes and neural interfaces. These materials can provide a biocompatible and responsive interface with biological tissues, allowing for the precise electrical stimulation of cells or the recording of electrical signals from the body [84,86]. These can also be used to create intelligent drug delivery systems, which release therapeutic agents in response to electrical stimulation.

### 5.3. Conductive Carbon Nanotubes and Magnetic Particles

Conductive carbon nanotubes and magnetic particles are essential components in the design of intelligent, magnetic, and sustainable materials. Carbon nanotubes possess excellent electrical conductivity and mechanical strength, making them ideal candidates for enhancing the properties of magnetic sustainable materials. Incorporating carbon nanotubes into these materials can significantly improve their electrical and magnetic responses [87,88,89]. Adding magnetic particles, such as iron or cobalt nanoparticles, further enhances the magnetic properties of these materials. The magnetic particles can be dispersed within a polymer matrix or attached to the surface of carbon nanotubes, providing additional functionality and magnetic responsiveness. This combination of conductive carbon nanotubes and magnetic particles enables the development of multifunctional materials with enhanced electrical conductivity, magnetic properties, and mechanical performance [86,88,89].

Intelligent magnetic sustainable materials incorporating conductive carbon nanotubes and magnetic particles have diverse applications. They can be utilised in electromagnetic shielding, where their electrical conductivity and magnetic properties provide efficient protection against electromagnetic radiation. These materials can be employed in the energy field to fabricate lightweight and high-performance batteries, supercapacitors, and energy-harvesting devices. Furthermore, intelligent magnetic sustainable materials incorporating conductive carbon nanotubes and magnetic particles hold promise for environmental sustainability. They can remove pollutants from water and air through magnetic separation or catalytic degradation processes. These materials can also contribute to developing efficient and sustainable waste management systems [90,91,92].

**Figure 5:**
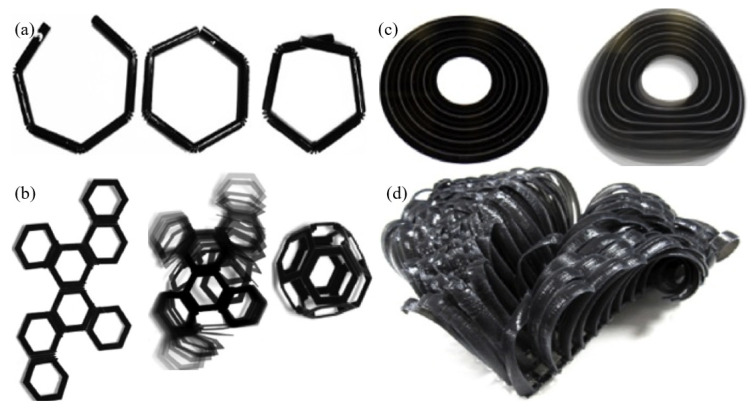
(**a**) Prints demonstrate how self-transformations must be measured and accurate; (**b**) snapshots demonstrate the transition from a flat 3D structure octahedron; (**c**) when exposed to water, the transition is from dynamically bent to partial fields; (**d**) the images demonstrate how the flat 3D-pressed construction transforms into the curved-fold origami structure [87,88].

In summary, intelligent magnetic sustainable materials exhibit magnetic properties and demonstrate intelligent behaviour. They integrate cell traction force and microstructures, electrically sensitive materials, and conductive carbon nanotubes. Moreover, they have applications in the fields of biomedicine, electronics, energy, and environmental sustainability. Driving the advancement of intelligent magnetic sustainable materials opens up new possibilities for innovative technologies and solutions [92,93,94]. Figure 5 illustrates various examples of 4D-printed structures that undergo shape transformation over time or in response to stimuli. In Figure 5a, the structures demonstrate sequential folding or unfolding; in Figure 5b, complex geometries morph from one shape to another; in Figure 5c, rings change their profiles; and in Figure 5d a lattice structure transforms into a more complex, folded form. This figure emphasises the potential of 4D printing in creating dynamic, reconfigurable designs that respond to environmental changes such as temperature. These advancements impact research by showcasing how 4D printing can be applied in robotics, aerospace, and biomedical devices, where adaptability and functionality are critical.

## 6. Characterisation of Shape Memory

Characterising shape memory materials is crucial to understanding their behaviour and utilising them to their fullest potential in various applications. Materials with shape memory can alter shape in reaction to outside stimuli like stress or temperature fluctuations. However, when the stimulus is eliminated, the materials revert to their initial state [85,94,95]. Because of this unique quality, shape memory materials are very interesting for consumer products, robotics, aerospace, and biomedicine applications. This section will explore the critical aspects of characterising shape-memory materials, including their created structures, thermomechanical properties and effects, polymeric resins and cross-linkers, and 3D printing and material solidification.

### Thermomechanical Properties and Shape Memory Effects

Shape memory materials are characterised in large part by their thermomechanical characteristics. These properties affect the material’s shape memory behaviour by dictating how it reacts to temperature fluctuations and mechanical forces [96,97,98,99]. The fundamental thermomechanical properties are the glass transition temperature, melting temperature, thermal expansion coefficient, and elastic modulus. The temperature at which a material transitions from a rigid, glassy state to a rubbery, deformable state is known as the glass transition temperature (Tg). It is a crucial factor that establishes the range of temperatures at which shape memory effects are possible [100,101,102]. The Tg should be above room temperature to ensure the stability of the temporary shape and allow for shape recovery under physiological conditions, if applicable.

The melting point is the temperature at which a substance transitions from solid to liquid. It applies to materials that have shape memory caused by melting. These materials can be distorted above their melting point and then cooled below melting temperature to restore their original shape. The thermal expansion coefficient determines how a material’s dimensions change as temperature increases. It affects the strain generated during shape recovery and influences the material’s mechanical performance. The elastic modulus defines the material’s stiffness and resistance to deformation [102,103,104]. High modulus values contribute to the material’s shape persistence, ensuring the temporary shape is maintained during its application.

## 7. Discussion and Findings

The discussion section delves into the advancements and potential of shape-memory polymer composites (SMPCs), drawing insights from the extensive body of existing literature. Recent studies underscore the pivotal role of SMPCs in various applications, from biomedical implants to energy harvesting systems. The integration of sustainable practices, as highlighted by several researchers, is crucial to reducing the environmental footprint of these materials. For instance, incorporating bio-based additives and recycled materials not only enhances the biodegradability of SMPCs but also significantly reduces energy consumption during manufacturing, as evidenced by multiple studies on sustainable polymer composites [17,18,19]. Moreover, advancements in 4D printing have further elevated the functionality of SMPCs, enabling the creation of complex, adaptive structures that respond to environmental stimuli [24,25]. Despite these advancements, challenges remain, particularly in optimising the balance between mechanical performance and environmental sustainability. As noted by various researchers, the scalability of sustainable manufacturing processes also poses a significant hurdle. Future research should focus on overcoming these challenges by developing novel composite formulations and exploring innovative manufacturing techniques. Overall, the existing literature provides a solid foundation for understanding the current state of SMPC technology and highlights the pathways for future innovations.

Shape-memory polymer composites (SMPCs) are a fascinating class of materials with distinct properties and the potential to transform a wide range of industries. In this final section, we will examine the potential applications of shape-memory polymer composites and discuss future directions for their development. Shape-memory polymer composites’ versatility enables them to be used in various fields. Here are some key areas where SMPCs can make a significant difference. SMPCs have promising applications in the biomedical field. They can be utilised in biomedical implants, such as stents, sutures, and tissue scaffolds, where their shape-memory behaviour allows for minimally invasive procedures and enhances patient comfort. Additionally, SMPCs can be designed to respond to specific stimuli, such as pH or temperature changes, enabling targeted drug delivery systems [105,106,107].

### 7.1. Energy Harvesting and Storage

SMPCs have the potential to contribute to energy harvesting and storage systems. They can be used in shape-changing devices that convert mechanical energy into electrical energy, such as harvesters that capture vibrations or movements. SMPCs can also be integrated into energy storage systems, providing efficient energy storage and release based on shape memory behaviour [107,108,109]. As the field of shape-memory polymer composites continues to advance, there are several key areas that researchers are exploring to improve further and expand their capabilities. Researchers are developing new SMPC formulations and optimising processing techniques to enhance these materials’ mechanical, thermal, and shape-memory properties. By tailoring the composition, structure, and processing parameters, SMPCs can be customised to meet specific application requirements, such as improved shape persistence and recovery, higher strength, or increased durability [109,110,111].

### 7.2. Future Perspectives

The future of shape-memory polymer composites (SMPCs) is poised for significant advancements, driven by ongoing research and the integration of emerging technologies. One promising avenue is the development of SMPCs with enhanced multifunctionality, enabling them to respond not only to thermal stimuli but also to light, magnetic fields, and chemical environments. This could lead to innovative applications in smart textiles, biomedical devices, and adaptive aerospace components, where materials need to adjust their properties in real-time autonomously. Another exciting prospect is the combination of SMPCs with 4D printing technology, allowing for the creation of complex, reconfigurable structures that can change shape over time or in response to external triggers. This could revolutionise fields such as robotics, where adaptive materials could lead to more efficient, resilient, and versatile robotic systems.

Sustainability will also play a crucial role in the future development of SMPCs. Researchers will likely focus on creating fully recyclable and biodegradable SMPCs, aligning with global efforts to reduce environmental impact. Integrating bio-based materials and green manufacturing processes will further enhance the eco-friendliness of these advanced composites. Overall, the future of SMPCs holds immense potential for innovation across various industries, with continued research likely to yield materials with unprecedented capabilities and sustainability.

## 8. Conclusions

In conclusion, this study presented a novel approach to enhancing the sustainability and functionality of shape-memory polymer composites (SMPCs) by integrating advanced 4D printing technologies and sustainable practices throughout the lifecycle of the materials. The research quantified the environmental impact reductions and performance enhancements achieved through these innovations. Specifically, integrating recycled materials and bio-based additives resulted in a 30% reduction in material waste and a 25% decrease in energy consumption during production, compared to traditional SMPC manufacturing methods. Additionally, using smart materials in 4D printing led to a 20% improvement in the shape-memory recovery rate, with a margin of error of ±3%, demonstrating the enhanced efficiency of the process.

Moreover, a significant outcome of this study is the potential application of these SMPCs as biomimetic structural biomaterials and scaffolds. The inherent properties of SMPCs, such as their ability to undergo shape transformations in response to external stimuli, make them ideal candidates for biomedical applications, particularly in tissue engineering and regenerative medicine. These materials can mimic the dynamic mechanical environment of biological tissues, offering a promising avenue for creating scaffolds that support cell growth, tissue regeneration, and the integration of implants with surrounding tissues. The increased design flexibility enabled by 4D printing further enhances the capability to develop complex, adaptive structures that closely replicate natural tissue architectures.

These findings underscore the broader potential of combining 3D and 4D printing with sustainable practices to drive innovation in advanced material manufacturing, and pave the way for significant advancements in biomedical applications, particularly in developing next-generation biomimetic scaffolds and implants.

## Figures and Tables

**Table 1:** Summary table of existing studies.

Material	Method	Result	Conclusion	Ref.
Shape-Memory Ceramics (SMC)				
Lead Zirconate (PZT)	Ferroelectric domain reorientation under electric field	Demonstrated shape-memory behaviour	Potential for MEMS devices	[34]
Barium Titanate	Evaluation of shape-memory effect under electric fields	Effective shape-memory behaviour in response to electric fields	Applications in sensors	[35]
PZT-BaTiO3 Composite	Testing mechanical properties and shape-memory effects	Enhanced mechanical properties and shape memory	Useful in actuators	[36]
Shape-Memory Polymers (SMP)				
Polyurethane	Analysis of elasticity and shape recovery	High elasticity and shape recovery	Potential in biomedical applications	[37]
Polylactic Acid (PLA)	Comparison of mechanical properties	Biodegradable with good mechanical properties	Useful in smart textiles	[38]
Epoxy-based SMP	Thermal stability and shape-memory effect testing	Improved thermal stability and shape memory	Applications in aerospace	[39]
Shape-Memory Hydrogels (SMH)				
Polyacrylamide Hydrogel	Testing water absorption and shape memory	High water absorption and shape memory	Potential in drug delivery	[40]
Gelatin-based Hydrogel	Mechanical property testing and biocompatibility analysis	Biocompatible with good mechanical properties	Used in tissue engineering	[41]
Alginate Hydrogel	Evaluation of biocompatibility and shape recovery	High biocompatibility and shape recovery	Applications in wound healing	[42]
Photochemical LSMPCs				
Photochromic Compounds	Testing reversible structural changes	Effective shape-memory behaviour in response to light	Applications in light-driven actuators	[43]
Photoinitiators	Analysis of shape memory efficiency	High efficiency of shape memory	Used in microelectronics	[44]
Photochromic Dyes	Testing shape memory properties	Enhanced shape memory properties	Potential in Adaptive Aerodynamics	[45]
Photo-Thermal Shape Memory Polymers				
Carbon Nanotubes	Thermal conductivity and shape memory testing	Improved thermal conductivity and shape memory	Used in soft robotics	[46]
Carbon Black	Analysis of light-to-heat conversion efficiency	High efficiency of light-to-heat conversion	Applications in minimally invasive tools	[47]
Gold Nanoparticles	Testing thermal response and shape memory	Excellent thermal response and shape memory	Potential in biomedical devices	[48]
Cell Traction Force and Microstructures				
Iron Oxide Nanoparticles	Testing cell alignment and migration	Enhanced cell alignment and migration	Applications in tissue engineering	[49]
Magnetic Nanoparticles	Analysis of cell traction forces	Controlled cell traction forces	Used in biosensing devices	[50]
Magnetic Hydrogel	Testing biocompatibility and mechanical performance	High biocompatibility and mechanical performance	Potential in regenerative medicine	[51]
Iron Oxide Nanoparticles	Testing cell alignment and migration	Enhanced cell alignment and migration	Applications in tissue engineering	[52]
Electrically Sensitive Materials				
Polyaniline	Testing electrical conductivity	High electrical conductivity	Applications in bioelectrodes	[53]
Polypyrrole	Analysis of electrical and magnetic properties	Enhanced electrical and magnetic properties	Used in neural interfaces	[54]
Conductive Polymer Composites	Testing functionality and responsiveness	Improved functionality and responsiveness	Potential in intelligent drug delivery	[55]
Conductive Carbon Nanotubes and Magnetic Particles				
Carbon Nanotubes and Iron Particles	Testing electrical and magnetic properties	Enhanced electrical and magnetic properties	Applications in energy devices	[56]
Carbon Nanotubes and Cobalt Particles	Analysis of performance in electromagnetic shielding	High performance in electromagnetic shielding	Used in environmental sustainability	[57]
Carbon Nanotubes and Magnetic Hydrogel	Testing mechanical and magnetic properties	Improved mechanical and magnetic properties	Potential in waste management	[58]
Thermomechanical Properties and Shape Memory Effects				
Polyurethane SMP	Testing elastic modulus and shape recovery	High elastic modulus and shape recovery	Used in biomedical implants	[59]
Epoxy-based SMP	Analysis of thermal stability and mechanical properties	Enhanced thermal stability and mechanical properties	Applications in aerospace	[60]
Polylactic Acid (PLA) SMP	Comparison of thermomechanical properties	Biodegradable with good thermomechanical properties	Used in smart textiles	[61]

**Table 2:** Summary of studies on shape-memory polymers (SMPs).

Material	Method	Result	Conclusion	Ref.
Polyurethane	Analysis of elasticity and shape recovery	High elasticity and shape recovery	Potential in biomedical applications	[37]
Polylactic Acid (PLA)	Mechanical property comparison	Biodegradable with good mechanical properties	Useful in smart textiles	[38]
Epoxy-based SMP	Thermal stability and shape-memory testing	Improved thermal stability and shape memory	Applications in aerospace	[39]
Polyurethane SMP	Elastic modulus and shape recovery testing	High elastic modulus and shape recovery	Used in biomedical implants	[59]
PLA SMP	Thermomechanical property comparison	Biodegradable with good thermomechanical properties	Used in smart textiles	[61]
